# Vulvar Cancer in the North of Israel

**DOI:** 10.5041/RMMJ.10156

**Published:** 2014-07-25

**Authors:** Orit Kaidar-Person, Nour Ibrahim, Amnon Amit, Roxolyana Bortnyak-Abdah

**Affiliations:** 1 Division of Oncology, Rambam Health Care Campus, Haifa, Israel and; 2 Gyneco-Oncology Unit, Rambam Health Care Campus, Haifa, Israel

**Keywords:** Chemotherapy, radiotherapy, surgery, vulvar cancer

## Abstract

**Purpose:**

This is a population study of patients who were treated for vulvar cancer in a tertiary center in northern Israel, aimed to report clinical findings, treatment, and outcome.

**Methods:**

A retrospective chart review of all medical records of consecutive patients who were treated for vulvar cancer in the years 1993–2012 was conducted. Data extracted from the medical records included demographics, histology, size of lesion, stage of disease at diagnosis, type of treatment, radiation dose, follow-up, recurrence, and survival.

**Results:**

The study included 44 patients with a median age of 69.8 years (range, 42–93 years). Thirty-five (79.5%) of the patients were of Jewish descent, five were Arabic, and four were of other descent. The most common histology type was squamous cell carcinoma in 35 (79.5%) patients. Most patients were staged FIGO II–III at time of diagnosis. Surgery was the most common primary treatment modality (54.2%). Twenty-three (52.2%) patients had recurrent disease. Older age and more advanced stage at diagnosis were associated with increased mortality.

**Conclusion:**

Vulvar cancer is common among elderly women with co-morbidities who present in advanced disease stage; all these factors are significant for survival.

## INTRODUCTION

Carcinoma of the vulva is a rare genital malignancy, accounting for 4% of all female genital malignancies, and up to 1% of all female cancers, occurring at a median age of 68–70 years.^1^,^2^ The great majority of cases (80%–90%) are squamous cell carcinomas, which also include two sub-classifications, basaloid and warty, more linked to human papillomavirus (HPV) than keratinizing squamous tumors.^3^ The HPV-associated subtypes are associated with younger age. The non-HPV-associated types are associated with chronic inflammatory states, such as lichen sclerosus. Smoking is also considered as a risk factor. Symptoms are not very specific and can present as itching, burning sensations, skin color changes, or bleeding. Moreover, about 50% of women with vulvar malignancy can be asymptomatic.^1^,^2^,^4^ Thus, diagnosis is often delayed.

Other histopathological subtypes of vulvar cancer include malignant melanoma (5%), basal cell carcinoma, Paget’s disease with or without adeno-carcinoma, Bartholin’s gland carcinoma, sarcoma, and other rare subtypes.^4^

The disease occurs primarily among older women (median age, 68 years), who often present at an advanced stage. This can be attributed to the asymptomatic nature of the disease or obscure symptoms (such as pruritus) and lack of awareness of the primary caregiver.^5^ Because vulvar cancer is rare and heterogeneous, and most epidemiologic and clinical data do not separate the subtypes and come from a limited number of cases analyzed retrospectively, compiled data must be interpreted cautiously. A previous report published over a decade ago summarized 50 cases of patients, treated over a period of 35 years (1961–1996) for squamous cell carcinoma of the vulva at Soroka Medical Center, the only tertiary medical facility in the south of Israel.^6^ Similar to Soroka Medical Center, our institution in northern Israel, Rambam Health Care Campus (RHCC), is a tertiary center and contains the only radiotherapy unit in the area. As a tertiary center, our patients often receive primary surgical treatment at other facilities and are referred to our center for radiotherapy and/or for primary treatment at our Gyneco-Oncology Unit. Our study aimed to report the clinical findings, treatment, and outcome for patients who were diagnosed with vulvar cancer and were treated in our Radiotherapy Unit during the past 20 years.

## METHODS

We conducted a retrospective chart review of all the medical records of consecutive patients who were treated in the Radiotherapy Unit at RHCC for vulvar cancer between the years 1993 and 2012. Data extracted from the medical records included demographics, histology, size of lesion, stage at diagnosis, type of treatment, radiation dose, recurrence, and survival.

## RESULTS

The study included 44 patients with a median age of 69.8 years (range, 42–93 years). Thirty-five (79.5%) patients were of Jewish descent, five were Arabic, and four were of other descent. Co-morbidities included hypertension in 26 (59%) patients, 14 (31.8%) had diabetes, and 10 (22.7%) had ischemic heart disease. Information regarding family history of malignancy was available for 39/44 patients; seven (17.9%) patients had first-degree relatives with various cancers. The primary clinical presentation was a vulvar lesion in 61.3%, pruritus in 27.2%, and 11% of the patients were asymptomatic. The most common histology type was squamous cell carcinoma in 35 (79.5%) patients (Table 1).

**Table 1.** Histology Subtypes.

**Histology**	**Number of Patients**
Squamous cell carcinoma	35 (79.5%)
Paget’s disease	3 (6.8%)
Primary mammary carcinoma	3 (6.8%)
Basal cell carcinoma	1 (2.2%)
Verrucous carcinoma	1 (2.2%)
Adenoid cystic carcinoma of Bartholin’s gland	1 (2.2%)

Only three patients were evaluated for HPV DNA, which was found to be positive in two. Four patients had a history of lichen sclerosus. Seven patients (of the 34 for whom the information was recorded) were smokers. Most patients (59.1%) were staged (via International Federation of Gynecology and Obstetrics staging system) as FIGO II–III at the time of diagnosis, and 9.1% were FIGO IV. Median tumor size was 3.14 cm (range, 0.12–7 cm), mostly located at the labia majora (77.3%) or clitoris (13.6%).

Surgical excision of the primary tumor with or without lymphadenectomy was the most common primary treatment modality (54.2%). Surgical procedures included wide local excision, radical vulvectomy, and modified radical vulvectomy, depending on stage at presentation. In only two (5.8%) patients who underwent surgery the surgical procedure include sentinel lymph node biopsy. Most patients who underwent lymphadenectomy had a bilateral procedure. Only three patients were reported to have gross positive margins, and the median margin size reported was 0.8 cm (0.1–1 cm) in the 20 cases that indicated the margin size in the pathology review.

Nine patients were treated with adjuvant radio-therapy (pelvic dose range, 30.6–50.8 Gy), while five were given a boost to tumor bed or positive margins (dose range, 10.8–21.6 Gy). Primary radio-therapy was given to 10 (27%) patients (pelvic dose range, 39.6–54 Gy), and seven were given a boost to gross tumor (dose range, 10–25.2 Gy). Field size, either in the adjuvant setting or as primary treatment, included small pelvis or whole pelvis with or without groin.

Chemotherapy alone was given in the adjuvant setting in one patient (5-fluorouracil and cisplatin) or concomitant with radiation (weekly cisplatin) in patients with gross positive margins or in patients who were treated by definitive chemo-radiation.

Adverse effects of radiotherapy (adjuvant and primary treatment) are recorded in Table 2. Adverse events that were recorded and related in the medical records to chemotherapy included severe hypomagnesemia in two patients; one patient who was treated with 5-fluorouracil and cisplatin suffered from chest pain during 5-fluorouracil infusion. Long-term toxicity included one case of rectovaginal fistula in a patient who was treated by surgery and adjuvant radiotherapy.

**Table 2.** Adverse Effects of Radiotherapy.

**Adverse Effects**	**Patients (%)**
Dermatitis at radiation field grade 3–4	42%
Diarrhea	15%
Cystitis	10.5%
Pain	5.2%

Twenty-three (52.2%) patients had recurrent disease; most (87%) were locoregional recurrences, and five cases were inside the radiation field. Median time to recurrence was 6.9 months (range, 4–21 months). Three of the patients who recurred suffered from deep vein thrombosis at time of recurrence.

Univariate analysis with use of the log-rank test of the variables age, stage, tumor size, family history of cancer, histology (squamous versus not), anatomic location, tumor histology, and grade indicated that older age and advanced stage at diagnosis were the only factors associated with increased mortality (*P*=0.018). Patients who underwent surgery had a median survival of five years versus patients who did not undergo surgery whose median survival was approximately one year (*P*=0.027).

## DISCUSSION

Even though our study is a retrospective one, it raises some important issues that need to be discussed. As previously stated, because vulvar cancer is heterogeneous, and because epidemiologic data often do not separate the subtypes, compiled data must be interpreted very cautiously.

As to the population in Israel, there is only one previous report published in an international journal on vulvar cancer in the south of Israel.^6^ Conclusions regarding demographics of these two reports should be taken with caution. However, both imply that vulvar cancer is more frequently diagnosed in the Jewish population than in the Arabic population.

Current data indicate that vulvar cancer, associated with HPV, occurs in up to 20% among women with primary cancers of the cervix and/or anogenital area, and the two diseases often occur simultaneously.^7^ In our study, only three patients were evaluated for HPV, and two were found to be positive. There were no cases of synchronous or metachronous anogenital cancer.

The median age of patients was 69.8 years, which also explains the prevalence of co-morbidities recorded in our patient group. This emphasizes an important clinical issue: the complexity of diagnosis and treatment. These patients were mostly diagnosed at an advanced stage, either because they were asymptomatic or because they had obscure symptoms, such as pruritus, which are often not related to cancer. Informing caregivers about a high level of suspicion in this population may enable diagnosis at an earlier stage. Early stage of disease will enable less extensive surgery without the need for adjuvant treatment. The extent of the surgical procedure is determined by the stage at diagnosis, co-morbidities, and the patient’s age and wishes.

The current trend is to perform less extensive procedures with no evidence of compromising outcome.^5^ A recent study published by Aragona et al. assessed independent prognostic factors according to data described in the literature in patients who were primarily treated by surgery (patients who underwent neoadjuvant therapy due to locoregion-ally advanced vulvar cancer were excluded).^2^ The authors identified a subgroup of patients considered as at a high risk for poor survival who may benefit from more aggressive intervention. Our study did not evaluate postoperative complications and mortality, as most patients were referred from different centers. Adjuvant treatment was given to patients with close or involved margins and/or positive lymph nodes. Adjuvant chemotherapy with cisplatin and 5-fluorouracil (active regimen against squamous cell cancer) inflicted chest pain during 5-fluorouracil infusion. Chest pain and ischemic complications are known to be rare adverse effects of treatment with various chemotherapy agents, mostly 5-fluorouracil, caused by various possible mechanisms.^8^ Since the patient population is older with co-morbidities, this should be taken into consideration, and proper monitoring is advised.

In our study, most patients who were inoperable were treated by chemo-radiation which was associated with treatment toxicity. There are no randomized trials comparing radiotherapy versus chemo-radiation as primary modality, but there is a preference for combined treatment.^5^ A recently published phase II study aimed to evaluate complete clinical and pathological response rates of the primary vulvar tumor to concomitant radiation (total dose of 57.6 Gy) with weekly cisplatin (40 mg/m^2^).^9^ The study included patients with locally advanced (T3 or T4) disease who were not candidates for surgical resection. Response was evaluated by surgical resection of the residual tumor or biopsy. Among 58 evaluable patients, 40 (69%) completed the study treatment, and 37 had a complete clinical response (37/58; 64%). Among these women, 34 underwent surgical biopsy and 29 (78%) also had a complete pathological response. Common adverse effects included pain, radiation dermatitis, and metabolic changes. In our cohort, severe acute dermatological complications were documented in 42% of the patients who were treated by radiotherapy. Currently, advanced radiotherapy treatment planning and irradiation techniques are available (such as intensity-modulated radiotherapy, IMRT) that may result in less skin toxicity in these patients. However, care must be given to allow full coverage of the target (skin); otherwise, the result will be greater local failure.

As noted in our cohort, although exclusively associated with recurrence, this population is at risk for deep vein thrombosis, extensive pelvic/groin surgery, and radiotherapy of the pelvis and groin, which distort the natural lymphatic drainage. In the study from Soroka Medical Center,^6^ eight patients suffered from leg lymphedema, two from leg lymph-angitis, and one from groin lymphocyst. Moreover, cancer and chemotherapy also contribute to a hyper-coagulable state. All these might imply that prophylactic anticoagulable treatment should be considered.

A recurrence rate of 52% is significantly high according to other published data. Other authors reported 75% recurrence-free survival rates. This is probably due to the fact that the data collected in this trial were of patients who were referred for radiotherapy due to advanced disease, thus, it is impossible to draw conclusions regarding these treatments and survival.

On a univariate analysis, older age and more advanced stage at diagnosis were the only factors found to be associated with increased mortality (*P* = 0.018). Patients who underwent surgery had a median survival of five years versus patients who did not undergo surgery, whose median survival was approximately one year (*P* = 0.027). This is probably biased, as patients who underwent surgery were in better medical condition and were at an earlier disease stage. Aragona et al. evaluated possible poor prognostic features by univariate and multivariate analysis.^2^ Factors evaluated included lymph nodes, tumor size, age, grade, lymphovascular space invasion, depth of stromal invasion, type of radical surgery, pathological margin distance, and stage. Multivariate analysis showed that the number of positive lymph nodes, extra-nodal growth, pathologic tumor size, and depth of stromal invasion proved to be independent prognostic factors. The authors also indicated that their study identified a high-risk group for failure to survive; this group included patients with tumor size 6–7.9 cm and depth of stromal invasion >4 mm, or tumor size ≥8 cm irrespective of depth of stromal invasion and extra-nodal growth, or ≥2 positive lymph nodes irrespective of tumor size and depth of stromal invasion.

## CONCLUSION

As indicated in our report, vulvar cancer is a rare disease, occurs mostly in elderly women, and is diagnosed at an advanced stage. Our findings emphasize that a greater effort should be made to facilitate early diagnosis, as treatment in earlier stages is less extensive and potentially curative.

## Abbreviations:

HPV: human papillomavirus;
RHCC: Rambam Health Care Campus.
